# Microbes in Infant Gut Development: Placing Abundance Within Environmental, Clinical and Growth Parameters

**DOI:** 10.1038/s41598-017-10244-x

**Published:** 2017-09-11

**Authors:** Tanja Obermajer, Iztok Grabnar, Evgen Benedik, Tina Tušar, Tatjana Robič Pikel, Nataša Fidler Mis, Bojana Bogovič Matijašić, Irena Rogelj

**Affiliations:** 1 Institute of Dairy Science and Probiotics, Biotechnical Faculty, University of Ljubljana, Domžale, Slovenia; 2 Faculty of Pharmacy, University of Ljubljana, Ljubljana, Slovenia; 3 Department of Gastroenterology, Hepatology and Nutrition, University Children’s Hospital, University Medical Centre Ljubljana, Ljubljana, Slovenia; 4 Group of Anthropology, Department of Biology, Biotechnical Faculty, University of Ljubljana, Ljubljana, Slovenia

## Abstract

Sound and timely microbial gut colonization completes newborn’s healthy metabolic programming and manifests in infant appropriate growth and weight development. Feces, collected at 3, 30, and 90 days after birth from 60 breastfed Slovenian newborns, was submitted to microbial DNA extraction and qPCR quantification of selected gut associated taxa. Multivariate regression analysis was applied to evaluate microbial dynamics with respect to infant demographic, environmental, clinical characteristics and first year growth data. Early microbial variability was marked by the proportion of *Bacilli*, but diminished and converged in later samples, as bifidobacteria started to prevail. The first month proportions of enterococci were associated with maternity hospital locality and supplementation of breastfeeding with formulae, while *Enterococcus faecalis* proportion reflected the mode of delivery. Group *Bacteroides-Prevotella* proportion was associated with infant weight and ponderal index at first month. Infant mixed feeding pattern and health issues within the first month revealed the most profound and extended microbial perturbations. Our findings raise concerns over the ability of the early feeding supplementation to emulate and support the gut microbiota in a way similar to the exclusively breastfed infants. Additionally, practicing supplementation beyond the first month also manifested in higher first year weight and weight gain Z-score.

## Introduction

Gut microbes in the early postnatal period play a significant role in a healthy child development. Sound and timely microbial stimuli during a nonrandom colonization process were addressed for their importance in the intestinal barrier development, activation and maturation of baby’s immune system, central nervous system development, metabolic system programming, and were finally linked to infant’s growth velocity and later life weight development^1–4^.

Fetal course of growth, set by genetic and environmental determinants at an early developmental phase, finally, results in the size at birth^5–7^. Various adverse life events, however, may interrupt the appointed infant’s growth trajectory. Regardless of an individual’s birth weight status, the accelerated postnatal growth was reported to unfavorably contribute to individual’s metabolic programming by subjecting him to an increased adiposity and to a risk for later life obesity^6, 8^. Namely, enhanced, unbalanced energy expenditure on growth at the cost of other developmental activities in their critical periods, was associated with later life chronic diseases^5^. During the postnatal maturation of various organ systems (metabolic, nervous, immune), in the neonate, brief reorganizational periods – windows, were suggested to overlap and interact with the time-specific and even species-specific microbial stimuli^1–3, 9, 10^. Alongside of diet- or gene-induced obesity, the microbe-induced obesity, driven by a compositional underrepresentation of the key protective intestinal taxa in the early life, was proposed^2^.

The susceptibility of the early microbial succession pattern to perinatal clinical and environmental circumstances was suggested to hold a remarkable potential to determine infant’s developmental fate and future well-being^11^. In European infants, differential environmental circumstances were suggested to affect gut microbiota development in compositional shifts following ‘north-south’ geographic gradient^12^.

Unique and immensely complex mature gastrointestinal tract (GIT) microbial communities result from the intrinsic microbial interactions and their developmental, host-associated determinants, which are still not satisfactorily understood^13^. Multivariate approach was suggested for the integrated investigation of the interactions among the microbial community members, environmental variables and individuals’ clinical aspects^14^. Moreover, longitudinal studies were reported to introduce the temporal developmental dynamics^15^.

Our longitudinal exploratory study aims to investigate the hypothesis that early and time-framed perinatal clinical events and environmental circumstances alter infant microbial gut colonization by affecting the abundances of specific microbiota members, which introduces changes in infant metabolic programming and reflects in later infant growth and development. We aimed to identify the factors that contributed substantially to infant microbiota shifts in Slovenian geographic locality, to relate them to infant growth and development during the first year, and finally, to determine the microbial shifts directly associated with infant growth and development at first year.

A comprehensive knowledge on the fecal microbial experience in the period of the microbiota acquisition and its relevance to the multiple early life exposures and clinical and developmental outcomes, would not only enable to identify the critical determinants of an ‘aberrantly’ colonized gut, but would also lead to the appropriate tailoring of the consortia in its initial, confined momentum. The clinical benefits of the microbiota targeted interventions during the colonization onset, however, could extend well beyond infancy, reducing the immense future health burden^4, 13, 16^.

## Results

The early life determinants for our study group are presented in details in Table 1. During the last course of pregnancy twelve women experienced the gestational diabetes or abnormal activity of the thyroid gland or asthma, but, no infections with antibiotic treatments were reported in the individuals prior birth. Preoperative intravenous antibiotic prophylaxis (single dose of cephalosporin) was administered in cesarean section delivery. After birth, health issues in six women were related to acute infections with antibiotic treatments. The use of two types of infant formulae was reported: Novalac (United Pharmaceuticals SAS, France) and Aptamil (Milupa Nutricia GmbH, Germany). Regular or occasional probiotic supplementation was recorded during the period of infant’s first three months. During the course of the study altogether ten infants experienced health complications related acute infections requiring antibiotic treatment or hospitalization (9) and hyperbilirubinemia at birth (1). The microbial representatives were analyzed in 167 fecal samples (day 3 postpartum (PP): 56 samples; day 30 PP: 57 samples; day 90 PP: 54 samples), and samples from all three time points were retrieved from 51 (85%) subjects.

**Table 1:** Demographic, clinical, environmental and anthropometric features of the mother-infant population in the study.

Perinatal variables	Population characteristic
Gender (M/F)	32/27 (54.2/45.8)
Gestational age (Full weeks)	39 [36, 41]
Mode of delivery (Cesarean section/Vaginal)	9/49 (15.5/84.5)
Maternity hospital - Regional (Murska Sobota, Kranj, Postojna) vs. Main (Ljubljana; Maribor)	15/44 (25.4/74.6)
Supplementation with commercially available probiotics containing lactobacilli (Yes/No)	14/44 (24.1/75.9)
Food Allergies during the first six months (Yes/No)	7/50 (12.3/87.7)
Residential environment (Rural/Urban)	18/39 (31.6/68.4)
Maternal age (Years)	30 [23, 44]
Maternal eduction (vocational/secondary/higher vocational/university degree/academic degree)	2/7/2/34/13 (3.4/12.1/3.4/58.6/22.4)
Maternal smoking prior pregnancy (Yes/No)	7/50 (12.3/87.7)
Length of breastfeeding (Months)	10 [3, 12]
Maternal body mass index before pregnancy	21.83 [18.08, 32.65]
Underweight/Normal weight/Overweight/Obesity	2/45/7/5 (3.4/76.3/11.9/8.5)
**Time specific determinants**	
Feeding type (Fully breastfed/Partially breastfed)	
30 days; 90 days	40/18 (69.0/31.0); 36/21 (62.1/36.2)
Infant’s health (Unhealthy/Healthy)	
Birth; 30 days; 90 days	3/54 (5.3/94.7); 5/52 (8.8/91.2); 7/50 (12.3/87.7)
Maternal health (Unhealthy/Healthy)	
During pregnancy; After birth	12/46 (20.7/79.3); 6/51 (10.5/89.5)
**Anthropometric data of newborns**	
Weight (g)	
Birth; 30 days;	3350 [2040, 4490]; 4645 [3190, 6210];
90 days; 1 year	6150 [4610, 8050]; 9590 [7520, 12610]
Body mass index (kg/m^2^)	
Birth; 30 days;	12.72 [10.54, 17.26]; 14.10 [10.82, 16.85];
90 days; 1 year	15.44 [11.80, 18.58]; 16.63 [13.53, 19.95]
Ponderal index (kg/m^3^)	
Birth; 30 days;	24.73 [21.08, 33.85]; 24.83 [18.66, 31.20];
90 days; 1 year	24.82 [18.88, 29.77]; 21.48 [17.91, 25.98]
Body fat (%)	
30 days; 1 year	12.0 [6.1, 20.3]; 11.1 [5.5, 22.9]
**Infants’ growth characteristics**	
Sex-standardized body mass index for age Z-score N < −0.67/−0.67 ≤ N ≤ 0.67/N > 0.67	
Birth; 30 days;	25/26/5 (44.6/46.4/8.9); 38/17/2 (66.7/29.8/3.5);
90 days; 1 year	37/13/7 (64.9/22.8/12.3); 19/24/13 (33.9/42.9/23.2)
Sex-standardized body mass index (BMI) for age Z-score change in individuals (i) between first/third/twelfth month and birth N < −0.67/−0.67 ≤ N ≤ 0.67/N > 0.67	
BMI Z_1i_-Z_0i_	23/25/8 (41.1/44.6/14.3)
BMI Z_3i_-Z_0i_	21/23/12 (37.5/41.1/21.4)
BMI Z_12i_-Z_0i_	8/25/22 (14.5/45.5/40.0)
Weight gain (WG) Z-score between months postpartum N < −0.67/−0.67 ≤ N ≤ 0.67/N > 0.67	
WG Z_(1–0)_	22/28/6 (39.3/50.0/10.7)
WG Z_(3–0)_	19/34/3 (33.9/60.7/5.4)
WG Z_(12–0)_	10/30/15 (18.2/54.5/27.3)
WG Z_(3–1)_	15/33/9 (26.3/57.9/15.8)
WG Z_(12–1)_	8/27/21 (14.3/48.2/37.5)
WG Z_(12–3)_	6/25/25 (10.7/44.6/44.6)

Continuous data are expressed as median [range], categorical as number (%) of subjects.

**Figure 1:**
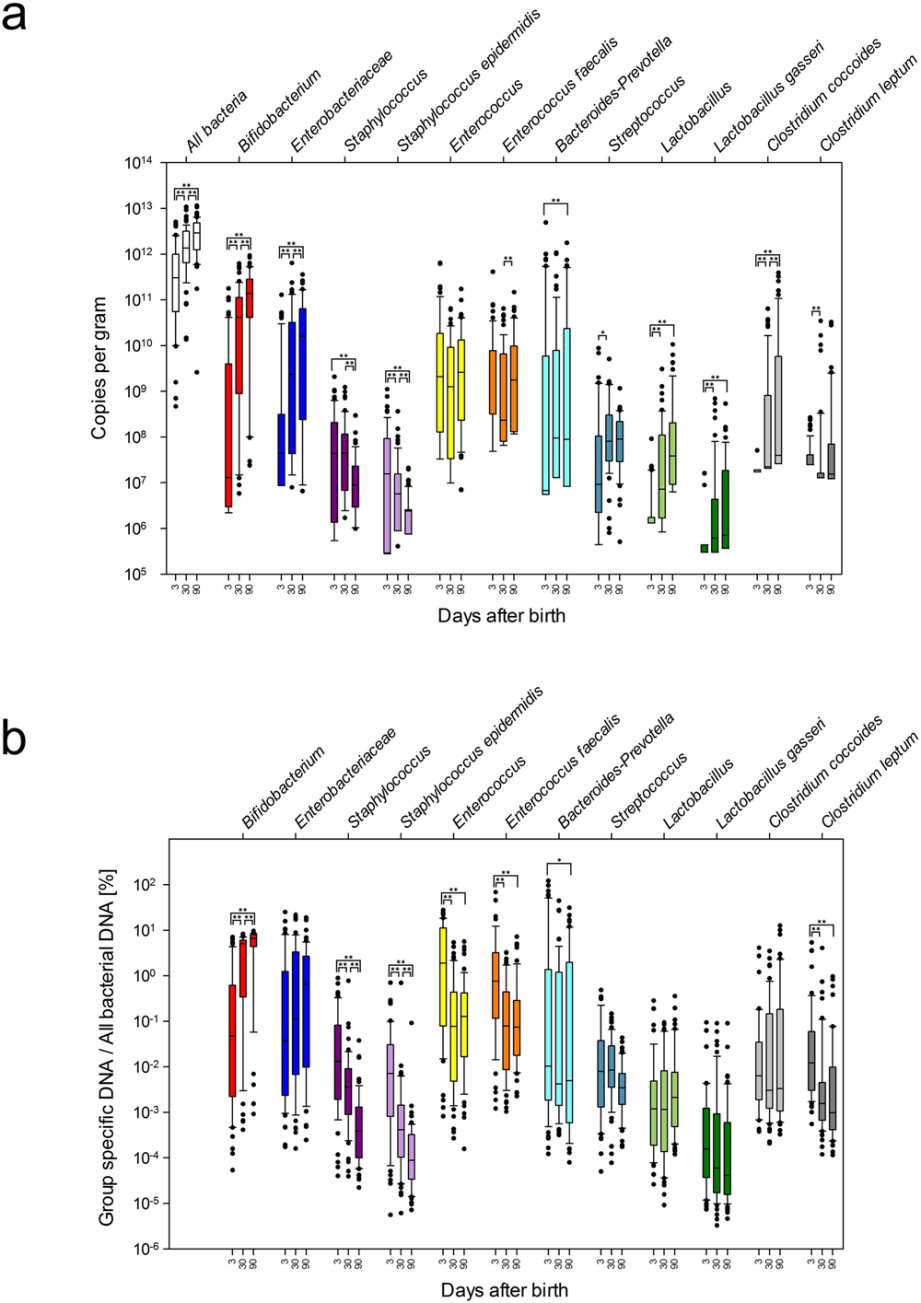
(**a**,**b**) Relative quantification of bacteria by real-time PCR in the feces of newborns in the first 3 months. The box and whiskers plots represent the medians and interquartile ranges; error bars 10^th^ and 90^th^ percentiles, filled circles outliers. Asterisks denote significant differences between time-points (*p < 0.05; **p < 0.01). The results are presented as a number of group specific bacterial DNA copies in g of feces (**a**), as ratio group specific DNA/all bacterial DNA (%) (**b**).

The formation of the initial gut microbiota in breastfed infants (≥36 gestational weeks) was characterized by an increased relative abundance of genus *Bifidobacterium* during the first three months, while relative abundances of staphylococci and enterococci simultaneously decreased (Fig. 1).

Samples, dominant in the class *Bacilli* (comprised of the cumulative relative abundance of staphylococci, streptococci, enterococci and lactobacilli), prevailed 3 days after birth (55%), while in the majority of later samples, *Bifidobacterium* group was recognized as the most abundant among the microbial groups surveyed.

Infants, classified on the basis of fecal microbiota composition into four groups as suggested by Dogra *et al*.^11^, are depicted in Table 2. The classification into four groups was made according to the prevailing microbial component (the highest relative abundance of a single group among the four compared; A- *Bacilli*, B- *Enterobacteriaceae*, C- *Bifidobacterium* and D- cumulative relative abundance of *Bacteroides-Prevotella* and *Clostridium* cluster XIV, IV) in the sample at specific time intersection. During the three months period the population drift towards the microbial profile C is discerned: 8.9% (5/56) of individuals reached profile C at the third day, 50.9% (29/57) by the end of the first month, and finally, 18.5% (10/54) only, by the end of the third month. By the end of the first month, the majority of subjects at third day placed in group A (21), and half of the subjects classified in group D due to highly abundant *Bacteroides-Prevotella* members (6), were caught in group C, rich in bifidobacteria while most of the subjects based in group B (6) and C (4) still lingered there. Four subjects stayed in group C for the whole trimester, and the additional 21 subjects joined in for the investigated last two months. During the last two months, however, limited number of individuals from the profiles A (1), B (4) and C (5) also progressed toward the profile D.

**Table 2:** Number (%) of samples classified according to the prevailing microbial component (containing the highest relative abundance among the four groups compared) at specific time intersection.

Age at sampling [days]	A- *Bacilli*	B- *Enterobacteriaceae*	C- *Bifidobacterium*	D- *Bacteroides-Prevotella* and *Clostridium* cluster XIV, IV
**3**	31 (55.4)*	8 (14.3)	5 (8.9)	12 (21.4)
**30**	6 (10.5)	12 (21.1)	33 (57.9)	6 (10.5)
**90**	1 (1.9)	5 (9.3)	35 (64.8)	13 (24.1)

Five samples (8.9%) at day 3 only, classified in group A (*), had none of the investigated microbial groups detected.

**Figure 2:**
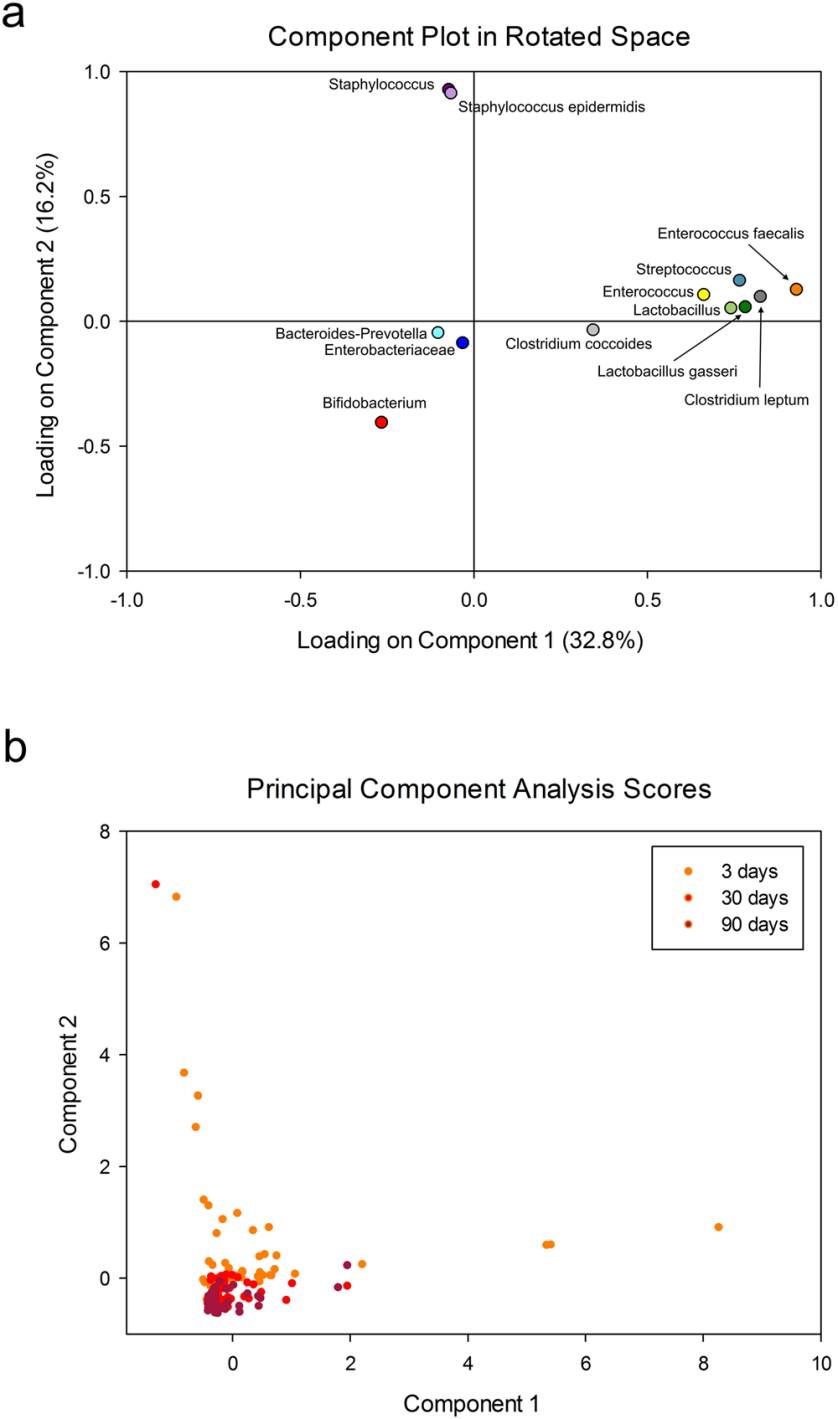
(**a**,**b**) Principal component analysis (PCA) with varimax rotation of the fecal microbiota in infants. Loadings of relative bacterial abundances (**a**). This figure shows the two primary principal components, which explain 32.8% and 16.2% of data variation. Individual PCA scores at 3, 30, and 90 days after birth (**b**).

With principal component analysis (PCA), two components were extracted, together explaining 49.0% (32.8% + 16.2%) of sample variance at all three collection time points. Bacterial groups described in Table 3 were associated with species *Enterococcus faecalis* (explaining the highest variability of the PC1 (0.93)) and genus *Staphylococcus* (explaining the highest data variation of the PC2 (0.93)). A loading plot is presented in Fig. 2a. Low and negative correlations with both components, however, were established for groups *Enterobacteriaceae*, *Bacteroides-Prevotella* and *Bifidobacterium*. Both principal components best explained the variability in initial microbial communities in subjects 3 days after birth, while in later sampling, this variability diminished (Fig. 2b).

**Table 3:** Sample prevalence and reaction parameters of qPCR investigated microbial groups.

Target organism	Prevalence of detected samples at day 3; 30; 90 [%]	Parameters of 6 qPCR runs			
		End cycle	R^2^ range	Efficiency [%] range	Mean LOD ± SD (copies per reaction mix; copies per gram feces)
All bacteria	100.0; 100.0; 100.0	30	0.998–0.999	103.1–105.5	224.4 ± 63.2; 4.5 × 10^7^ ± 1.3 × 10^7^
*Bifidobacterium* group	57.1; 94.7; 100.0	30	0.991–0.998	93.4–96.9	38.8 ± 18.7; 7.8 × 10^6^ ± 3.7 × 10^6^
*Enterobacteriaceae* group	28.6; 80.7; 87.0	30	0.995–0.999	96.1–101.8	152.3 ± 146.6; 3.0 × 10^7^ ± 2.9 × 10^7^
*Staphylococcus* group	76.8; 82.5; 81.5	30	0.995–0.999	88.6–93.7	12.6 ± 7.9; 2.5 × 10^6^ ± 1.6 × 10^6^
*Staphylococcus epidermidis*	71.4; 68.4; 31.5	30	0.995–0.999	94.2–99.2	8.4 ± 8.0; 1.7 × 10^6^ ± 1.6 × 10^6^
*Enterococcus* group	55.4; 79.0; 94.4	30	0.998–1.000	90.9–94.7	331.4 ± 475.7; 6.6 × 10^7^ ± 9.5 × 10^7^
*Enterococcus faecalis*	46.4; 52.6; 74.1	30	0.993–0.999	77.4–82.1	1291.0 ± 971.4; 2.6 × 108 ± 1.9 × 108
*Bacteroides-Prevotella* group	33.9; 33.3; 35.2	30	0.996–0.999	96.7–100.0	360.6 ± 430.0; 7.2 × 10^7^ ± 8.6 × 10^7^
*Streptococcus* group	57.1; 100.0; 98.2	30	0.996–1.000	90.0–93.5	6.9 ± 7.7; 1.4 × 10^6^ ± 1.5 × 10^6^
*Lactobacillus* group (including *Leuconostoc, Pediococcus, Weisella)*	21.4; 56.1; 59.3	30	0.997–0.999	80.8–83.9	35.1 ± 34.3; 7.0 × 10^6^ ± 6.9 × 10^6^
*Lactobacillus gasseri*	5.4; 26.3; 33.3	28	0.994–0.999	96.8–100.9	4.5 ± 1.7; 9.1 × 10^5^ ± 3.4 × 10^5^
*Clostridium coccoides* group (cluster XIVa and XIVb)	1.8; 28.1; 44.4	30	0.996–0.999	85.5–88.4	241.6 ± 81.7; 4.8 × 10^7^ ± 1.6 × 10^7^
*Clostridium leptum* group (cluster IV)	12.5; 15.8; 29.6	30	0.995–0.998	89.9–94.2	202.8 ± 108.6; 4.1 × 10^7^ ± 2.2 × 10^7^

Bivariate analyses of the associations of microbial abundances with clinical, environmental, anthropometrical variables, and finally, with infant’s growth deviation scores have been performed. Multiple linear regression followed on the constricted set of the associated (p < 0.1) independent variables. The established associations are presented (Table 4, Supplementary Table S1(a,b)).

**Table 4:**
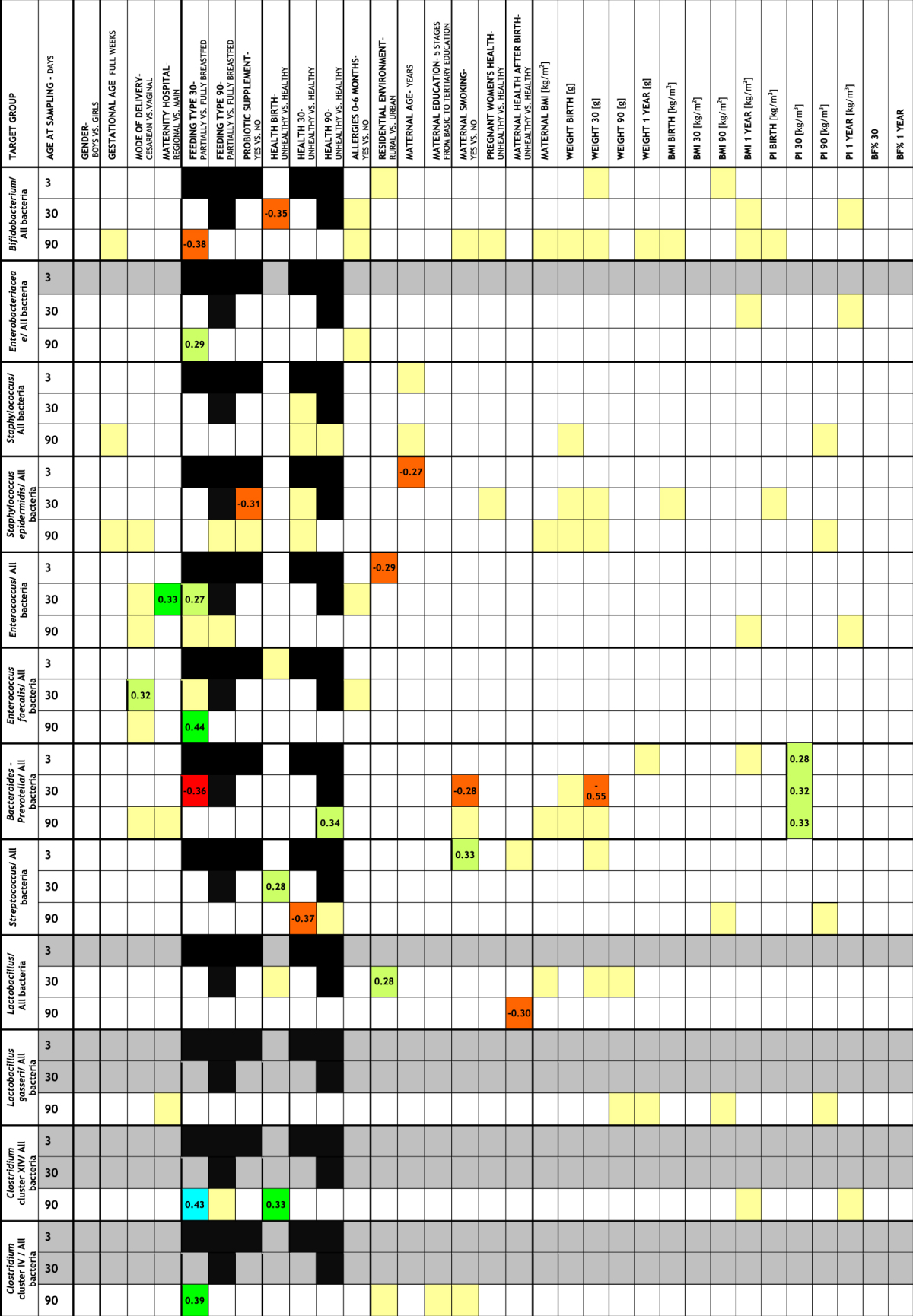
Multiple regression analysis of the associations of logit transformed relative abundances of microbial groups with demographic, environmental, clinical, and anthropometric characteristics of the mother-infant pairs. Only significant associations (p < 0.05) are presented. Predictor variables included in the regression model (yellow fill) were selected by *non-parametric* bivariate analyses of the relative microbial abundances and the independent variables. Criterion for the inclusion in the model was (p < 0.1). Furthermore, multiple regression analysis revealed no association between microbial abundances and BMI Z-score changes and weight gain Z-scores. The color intensity indicates the strength and direction of the association, while numbers in the boxes indicate the standardized effect sizes. Bacterial groups with <30% detected samples (grey fill) and irrelevant associations (black fill) are indicated. Positive associations presented in turquoise fill (p < 0.001), green fill (p < 0.01), light green fill (p < 0.05) and negative associations in orange fill (p < 0.05) and red fill (p < 0.01).

After adjustment for potential confounders, maternity hospital (regional (15) vs. main (44)) reflected in higher relative abundance (%) of fecal enterococci at first month (median (m): 6.0 × 10^−1^, range (r): 9.2 × 10^−4^–7.7 vs. m: 7.8 × 10^−2^, r: 1.6 × 10^−4^–2.2 × 10^1^; p = 0.008). Relative abundance of *Enterococcus faecalis* at first month was higher in infants born via C-section (9) compared to those vaginally delivered (49) (m: 6.7 × 10^−1^, r: 1.3 × 10^−2^–3.2 vs. m: 3.5 × 10^−2^, r: 1.0 × 10^−3^–2.1; p = 0.020). The comparison of absolute and relative abundances of microbial representatives in fecal samples from partially (18) versus exclusively (40) breastfed infants at first month across the three time intersections are presented in Supplementary Fig. S1 and Supplementary Fig. S2. Interestingly, in multivariate analysis no association of microbiota in fecal samples at 90 days was observed with the feeding status after the first month (Table 4).

Lower proportions of *Staphylococcus epidermidis* (m: 7.5 × 10^−5^, r: 6.1 × 10^−6^–2.7 × 10^−3^ vs. m: 6.4 × 10^−4^, r: 1.5 × 10^−5^–7.0 × 10^−1^; p = 0.044) at the end of the first month, distinguished babies consuming probiotic preparation (14) from those, who did not (44). This bacterial group was in starting samples also inversely correlated with maternal age (Spearman’s rho (r_s_): −0.328; p = 0.016).

Subjects experiencing health complications at birth (3) diverged from the majority of healthy individuals (54) by relative abundance of *Bifidobacterium* (m: 2.8 × 10^−2^, r: 6.3 × 10^−4^–1.6 × 10^−1^ vs. m: 5.1, r: 4.1 × 10^−4^–8.3; p = 0.011) and *Streptococcus* (m: 9.4 × 10^−2^, r: 1.5 × 10^−2^–1.2 × 10^−1^ vs. m: 8.2 × 10^−3^, r: 7.7 × 10^−5^–1.5 × 10^−1^; p = 0.042) at one month, and *Clostridium* cluster XIV (m: 7.2 × 10^−1^, r: 1.6 × 10^−1^–2.4 vs. m: 3.0 × 10^−3^, r: 3.3 × 10^−4^–1.3 × 10^1^; p = 0.004) at third month.

Health complications reported for five infants at the end of the first month, were in comparison to healthy subjects (52) associated with a shift in *Streptococcus* at third month (m: 5.5 × 10^−4^, r: 1.9 × 10^−4^–2.4 × 10^−3^ vs. m: 3.8 × 10^−3^, r: 1.8 × 10^−4^–4.4 × 10^−2^; p = 0.015), and finally, seven babies with health issues recorded at third month compared to the healthy subpopulation (50), were associated with higher proportion (%) of *Bacteroides-Prevotella* (m:1.5 × 10^1^, r: 1.5 × 10^−4^–3.1 × 10^1^ vs. m: 2.4 × 10^−3^, r: 7.9 × 10^−5^–1.2 × 10^1^; p = 0.025) in samples at third month.

Maternal antibiotic treatments after birth characterized six infants at the end of their third month, compared to those, whose mothers were not exposed to antibiotics (51), by lower proportion (%) of *Lactobacillus* (m: 3 × 10^−4^, r: 1.2 × 10^−4^–5.3 × 10^−3^ vs. m: 3.4 × 10^−3^, r: 1.4 × 10^−4^–3.6 × 10^−1^; p = 0.031). Higher relative abundance of lactobacilli at the end of the infants’ first month was associated with rural maternal residence (18) compared to those from the urban background (39) (m: 5.2 × 10^−3^, r: 1.6 × 10^−5^–9.4 × 10^−2^ vs. m: 4.3 × 10^−4^, r: 9.1 × 10^−6^–8.5 × 10^−2^; p = 0.029). Urban maternal background was associated with higher initial enterococci ratio (%), compared to infants born to mothers from rural settings (m: 2.7, r: 1.3 × 10^−3^–2.8 × 10^1^ vs. m: 2.9 × 10^−1^, r: 1.8 × 10^−3^–2.4 × 10^1^; p = 0.033). Maternal smoking prior pregnancy (7) was associated with higher proportion of streptococci in the offspring samples three days after birth compared to samples from babies born to non-smokers (50) (m: 8.4 × 10^−2^, r: 3.5 × 10^−3^–4.8 × 10^−1^ vs. m: 5 × 10^−3^, r: 1.2 × 10^−4^–3.5 × 10^−1^; p = 0.020) and lower proportion of *Bacteroides-Prevotella* in the samples obtained at the end of the first month (m: 1.4 × 10^−3^, r: 3.4 × 10^−4^–4 × 10^−3^ vs. m: 6.7 × 10^−3^, r: 3.2 × 10^−4^–4.5 × 10^1^; p = 0.031).

Relative abundances of *Bacteroides-Prevotella* group in the samples at 3, 30, and 90 days were associated with ponderal index (PI) at one month (r_s_ = 0.27, p = 0.041; r_s_ = 0.23, p = 0.022; r_s_ = 0.23, p = 0.048, respectively). Finally, first month *Bacteroides-Prevotella* sample content was also inversely associated with infant’s first month weight (r_s_ = −0.28, p = 0.028). The opposite direction of the observed associations with PI and weight was attributed to the negative associations of this bacterial group with height. It should also be noted here that no association with early BMI was found. However, none of the studied microbial groups was associated with growth indicator deviation scores.

Additionally, bivariate associations established for the bacterial groups with low sample prevalence (<40%) (Table 3) were investigated in the logistic multiple regression analysis (Supplementary Table S1(a,b)). If infant weight gain Z-score 12 months −3 months (WG Z_(12–3)_) was lower (odds ratio (OR) = 0.30, p = 0.032), if maternal pre-pregnancy body mass index (BMI) was higher (OR = 1.25, p = 0.040), and finally, if infant% body fat (BF%) at 1 year age was higher (OR = 1.57, p = 0.023), probability for *Clostridium* cluster XIV detection in babies at one month of age was higher. Also, likelihood for *Lactobacillus gasseri* detection in stools of 3 months old babies was higher, if maternal BMI was higher (OR = 1.30, p = 0.029).

**Figure 3:**
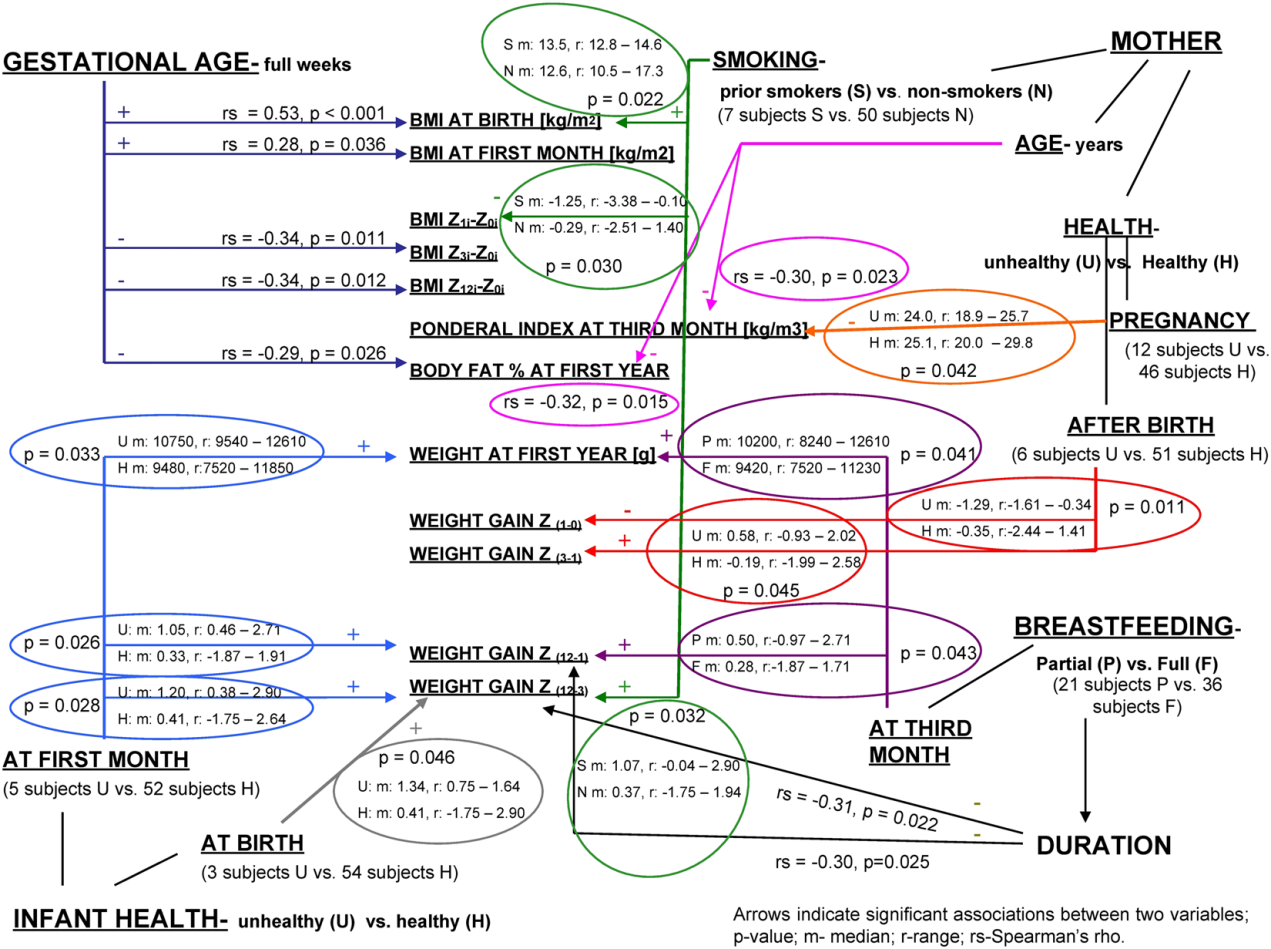
Diagram presenting values for the exposed significant associations between the infant growth parameters and presumptive perinatal growth confounders.

Limited associations found between bacterial proportions and anthropometrics with growth related data led us to investigate the presumptive perinatal growth confounders (results from bivariate analyses are presented in Table 5 and Fig. 3). Gestational age correlated positively with BMI at birth and first month and negatively with the difference in BMI Z-scores (Z_3i_-Z_0i_; Z_12i_-Z_0i_) and BF% at first year. Compromised infants’ health at birth was associated with the WG Z_(12–3)_, while health related issues at 1 month of age were associated with higher weight (g) at 1 year and higher WG Z_(12–1)_ and WG Z_(12–3)_. Subjects on supplemental formula feeding after the first month were positively associated with WG Z_(12–1)_ and weight at 1 year compared to fully breastfed, while negative associations were established for duration of breastfeeding with WG Z_(12–1)_ and WG Z_(12–3)_. Infants whose pregnant mothers experienced health issues compared to healthy mothers had lower PI at third month PP (PI 90), while antibiotic treatment of mothers after birth was related with lower WG Z_(1–0)_ and higher WG Z_(3–1)_ compared to healthy subpopulation. Infants born to prior smokers had higher BMI at birth and were negatively associated with BMI Z score change from birth to 1 month (BMI Z_1i_ - Z_0i_) and positively associated with WG Z_(12–3)_ compared to non-smokers. Infants born to elder mothers had lower PI 90 and lower BF% at first year.

**Table 5:**
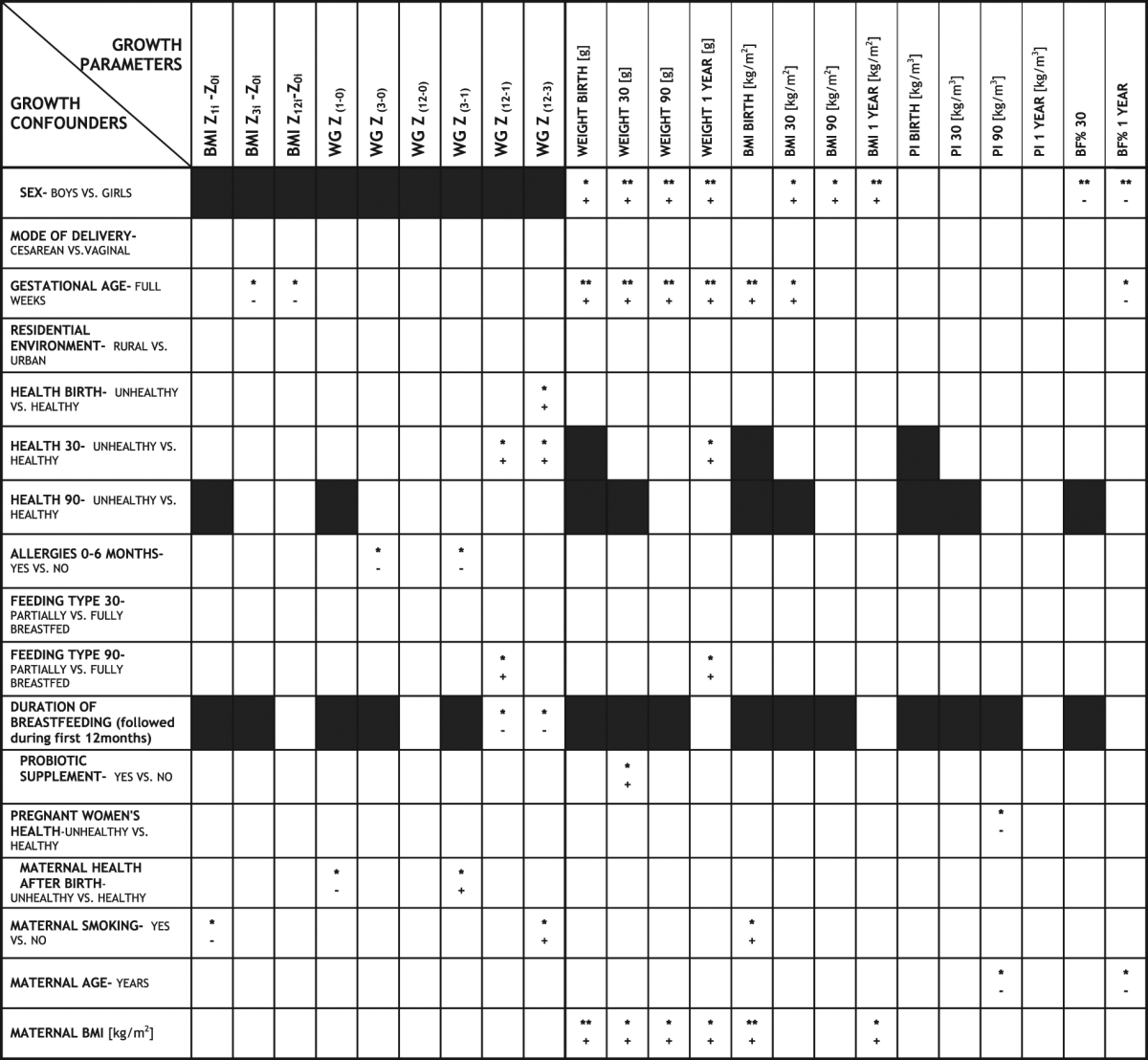
Bivariate non-parametric associations of growth parameters with growth confounders. Direction (positive for increase and negative for decrease) of the significant associations *(p < 0.05) and **(p < 0.01) are presented. Irrelevant associations are indicated with black fill.

Finally, for the mode of delivery and residential environment no associations with anthropometric or growth parameters were established.

## Discussion

The successional evolution of microbes residing in the gut of neonates, surveyed in our study, generally resembled the colonization stages proposed by Dogra *et al*.^11^. However, the microbial profiles established 3 days after birth, prevailed in bacterial groups from the class *Bacilli*, thought to be a common characteristic for the premature infants. Interestingly, *Bacilli* represented the largest proportion of the colostrum microbial communities in the subpopulation of lactating Slovenian mothers^17^. A phenomenon of reduced and delayed gut colonization by Gram-negative bacteria (enterobacteria and *Bacteroides*) with an increased Gram-positive initial bacterial load (staphylococci, enterococci, bifidobacteria and clostridia) was reported to reflect the increased hygiene in the Western countries^18^. Cross-sectional study by Fallani *et al*.^12^ exposed higher fecal proportions of bifidobacteria in six weeks old infants and lower proportions of *Bacteroides*, enterobacteria and lactobacilli to be typically associated with the North European countries. Term, breastfed Slovenian infants reached approximately 1–2 logs higher average counts of fecal bifidobacteria compared to twenty term, exclusively breastfed infants from Northern Spain^19^. Our results are in line, though, with the prevailing bifidobacterial counts detected in one month old infants from KOALA Birth Cohort Study, conducted a decade ago in Netherlands^20^. During the first three months, *Bacteroides-Prevotella* group was detected only in one third of samples from Slovenian infants, but the average counts were higher 1–2 logs compared to the study by Arboleya *et al*.^19^. Average values for the lactobacilli at first month and enterobacteria during the period of three months, in our study, were comparable to Spanish infants.

Within the first six months of life, an earlier acquisition of an anaerobic microbiome, dominated by *Bifidobacterium* and *Collinsella*, was found associated with typical adiposity at 18 months of age^21^. In our study, however, direct links between the proportions of surveyed microbes in the samples during the first three months and infant adiposity (measured as BF% at first month and at first year) were not established. But, BF% at the end of the first year was negatively associated with male gender (p < 0.001), maternal age (r_s_ = −0.321, p = 0.015) and gestational age (r_s_ = −0.294, p = 0.026). Even though gestational age was proposed to chiefly dictate the tempo of microbial development in the gut of premature infants^22^, within our set of gestational periods, no divergences in the proportions of the acquired early microbes were established in multivariate analysis. Nevertheless, higher maternal age was associated (p = 0.046) with lower relative abundance of *Staphylococcus epidermidis* in infant’s feces 3 days after birth, and apart from lower BF% at first year, also with lower PI 90 (r_s_ = −0.297, p = 0.023). Our observations on an early infant phenotype support the recent study on the socio-economic homogenous group of participants, linking higher maternal age at birth with taller size and the reduced abdominal fat in the respective pre-pubertal children^23^. The reported associations could originate from various prenatal factors (genetic, epigenetic, physiological- maternal hormonal changes), or variation in colostrum/milk composition^24–26^.

Enterococci colonize vaginally and C-section delivered infants equally early^18^. While reports indicated that meconium samples harbored also many *Enterococcus faecalis* isolates, some of these early colonizers were even found bacteriocin producers and were suggested as probiotic candidates^27, 28^. In our analysis, higher relative abundance (p = 0.020) of *Enterococcus faecalis* one month after birth only, marked samples of C-section infants, though, this difference dissipated in later samples. Except for this variation, other examined ‘developmental taxa’ remained quantitatively unaffected. Similarly, regional maternity hospitals induced shifts towards higher enterococci (p = 0.008), but transiently, only in samples one month after birth.

Jakobsson *et al*.^29^, however, observed C-section to be associated with delayed colonization, lower abundance and diversity of *Bacteroidetes* phylum in infant intestinal microbiota during the first two years of life. Recent meta-analysis^30^, founded on the reviewed epidemiological evidences, supported a higher risk of developing childhood obesity for children delivered by caesarean section, even after the adjustment for maternal pre-pregnancy weight as a potential confounder. The bias for the residual confounders (gestational age, breastfeeding, gestational diabetes, birth weight, etc.) on the obtained results, however, could not have been excluded. In our study, delivery mode did not induce any variations associated with the inspected growth and anthropometric parameters within the infants’ first year of life. Yet, *Bacteroides-Prevotella* relative abundance in one month old infants was found inversely associated with infant’s first month weight (p = 0.028), formula supplementation (p = 0.004), and prior maternal smoking (p = 0.031), while positive association for this bacterial group was demonstrated with PI at first month (PI 30) in samples from all three time intersections (p = 0.041; p = 0.022; p = 0.048). During the period of the first three months, however, this microbial member was detected in only small percentage of samples, therefore, further investigation on its potential to uphold a protective role in the newborns’ gut is warranted. Riva *et al*.^31^ explained that lower levels of *Bacteroidetes* taxa typically found in the gut microbiota of obese children may stem from the phylum ‘intra-family’ ecological cohesion, involving similar niche responses of microbiota members which positively correlate with one another, and thus, make this phylum a better forecaster of obese phenotype than *Firmicutes* lineage^31^. Though, certain *Firmicutes* populations on the lower taxonomic scale were reported to be associated with childhood obesity.

In search for a time-dependent early gut stimuli associated with the expected growth in infants, White *et al*.^32^ examined the detection of the probe signals in the fecal samples of term, vaginally born Norwegian infants, initially not exposed to antibiotics. Among different microbial groups and time points surveyed within the first four months of life, growth outcomes were associated with staphylococci, *Escherichia coli* and *Bacteroides* species. Following, it was also reported that the presence of *Bacteroides fragilis* group in stools of 1 month old infants was likely related to higher BMI later in childhood^33^. Altogether, based on the reviewed literature data, involvement of staphylococci, higher lactobacilli, lower *Bacteroides* spp. and bifidobacteria in gut colonization within the 3 months of birth, were hypothesized to be predictive for infant and child overweight^34^. In our study, after a multiple linear regression, except for the relative abundance of *Bacteroides-Prevotella*, other bacterial groups were not found directly associated with any of the investigated infant anthropometric measurements nor growth deviation scores during the first year. Though, the most versatile microbial associations with the external parameters, related to the pace of the ‘evolutionary taxa colonization’, in our study, were found with respect to infant early feeding pattern and early health.

Relative abundances of bacterial genera in the three months old infants were reported to be affected by formula type of feeding, resulting in a lower *Bacteroides* and higher *Firmicutes* (*Clostridium* XVIII, *Lachnospiraceae*, *Streptococcus*, *Enterococcus* and *Veillonella*)^35^. Mixed feeding pattern, in the study by Penders *at al*.^20^, did not affect fecal bifidobacteria at first month. Still, report scarcity on the early mixed-feeding practices and the related infants’ clinical outcomes^36^ make our contribution a valuable extension. Our data on the mixed-feeding practice in one month old infants were associated with lower fecal relative abundance of *Bacteroides-Prevotella* group (p = 0.004) and higher enterococci (p = 0.036), while delayed associations extended also further to samples from three months old infants having lower relative abundances of bifidobacteria (p = 0.011) and higher relative abundances of *Enterobacteriaceae* group (p = 0.039), *Enterococcus faecalis* (p = 0.002), and both investigated clostridia groups (p < 0.01). As suggested^35^, these differences most likely result from the capability of bifidobacteria and *Bacteroides-Prevotella* group to utilize human milk oligosaccharides (HMO), while also being capable to metabolically cross-feed other gut members from the *Firmicutes* lineage. Moreover, the breast milk oligosaccharide array – sialylated HMO, were recently reported to correlate with infant growth outcomes via the microbiota-dependent metabolic route and also promoted phenotypes with an improved usage of dietary components^37^. However, no associations were found on the microbes in the feces at the age of three months and the relevant feeding data for the corresponding two-month period, which could reflect the importance of timing on the feeding variable to exert its influence on the microbiota. Additionally, the fact that some mothers one month PP reversed the type of baby’s feeding - either they started to nourish their neonate solely on breastmilk (five) or began to supplement their nutrition with formula (nine), may have blurred the differences in the microbial representatives between both investigated groups. Practiced feeding type, however, was not submitted to prior infant growth and anthropometrics as affirmed by lack of the established associations. As reported previously, initial breastfeeding led to faster weight gain in the first few months compared to formula feeding, whereas only later on, breastfed infants demonstrated lower weights^38^. In accordance, in our study, exclusive breastfeeding pattern only after first month was found associated with lower WG Z_(12–1)_ (p = 0.043) and weight at first year (p = 0.041). In addition, duration of breastfeeding was negatively associated with the WG Z_(12–1)_ and WG Z_(12–3)_ (r_s_ = −0.299, p = 0.025 and r_s_ = −0.306, p = 0.022, respectively). Bäckhed *et al*.^39^ emphasized also the role of nutrition in the first year microbiota maturation and function and exposed the cessation of breastfeeding rather than the dietary integration of solid foods to be associated with microbial configuration shifts. We would like to note here, that the duration of breastfeeding in our population was also associated with infant feeding pattern at third month (p < 0.001). Namely, exclusively breastfed infants were breastfed for a longer period during the first year than partially breastfed peers. Additionally, lower birth weight was associated with higher BMI Z_1i_-Z_0i_, BMI Z_3i_-Z_0i_, and BMI Z_12i_-Z_0i_ (p < 0.01), but not with WG Z-scores.

In our study, infants experiencing health issues at birth and during first month, manifested also higher WG Z-scores (p < 0.05). Even though relative abundances of single microbes were not directly associated with growth parameters, the health variables at the observed time-intersections were found extendedly associated with differential proportions in some microbial groups. The associations were mostly discerned with a lag, at a later time intersections. Similarly, in these subjects the time lag in differential weight gain also manifested later, during the period of the first year. The ‘growth stimulating’ effect associated with the health status at birth and at first month of age could have resulted from the antibiotic treatments, which is in accordance with the recent publications^40, 41^. Moreover, it was suggested^40^ that the far-reaching repercussions of the antibiotics on the fecal microbial composition in the pre-school children could linger for up to two years after refraining from the antibiotic usage. Though, higher relative abundance of *Bacteroides-Prevotella* group (p = 0.025) established in the ‘unhealthy’ seven babies at three months of age, did not mirror any growth deviations in the first year of life.

Maternal lifestyle concerning her residential environment and health also reflected in several compositional shifts. We argue these associations with the infant fecal microbiota could have been introduced through differential composition of the maternal colostrum and breast milk. Inclinations for higher median counts of total cultivable lactobacilli in breast milk of women living in the rural areas compared with those from urban areas were also reported previously^42, 43^. Moreover, lactobacilli and bifidobacteria were less frequently found in the breast milk of women on prior antibiotic therapy during pregnancy and lactation^44^. While in our study, maternal postnatal antibiotic consumption similarly reflected in lower relative abundance of fecal lactobacilli in 3 months aged infants (p = 0.031), also infant WG Z_(1–0)_ and WG Z_(3–1)_ during the lactation period were affected (p = 0.011 and 0.045, respectively). Smoking cessation was proposed to induce changes in the composition of the intestinal microbiota similar to the differences reported between obese and lean humans and was also proposed to influence the individual’s weight gain^45^. Even though all mothers but one ceased smoking during pregnancy, the extended associations found in the initial microbial colonizers in their offsprings could have been introduced via the entero-mammary pathway^46^ or enhanced via the epigenetically modified compounds in breast milk^47, 48^. More importantly, however, seven infants born to prior-smokers, also demonstrated higher BMI at birth (p = 0.022), lower BMI Z_1i_-Z_0i_ (p = 0.030) and higher WG Z_(12–3)_ (p = 0.032), which even exceeded + 0.67 cut-off in five infants and thus may predispose them to later obesity risk^8^. No pre-/during-/post-pregnancy maternal BMI associations were established, however, between priorly smoking and non-smoking mothers.

Limited population size, low sampling intensity and the restricted microbial survey may have affected the resolution potential of our research, presenting the colonization progression in the gut of mature Slovenian infants in association with the related confounders. We also realize, early compositional deviations found in some subjects may have originated from the unpredictable events or sudden shifts. We would like to remind, therefore, to keep the results bearing the unproportionate and small groups of individuals, especially those concerning health variables, in perspective.

## Conclusions

Fecal microbiota at third day postpartum in term Slovenian breastfed infants prevailed in bacterial groups belonging to the class *Bacilli*, which was thought to be a common characteristic for the premature babies. Bifidobacteria, however, became dominant in the majority of samples later, at first and third month. Also, the variability in microbial proportions was noteworthy in these early, initial samples, especially for the early proportional content of *Enterococcus faecalis* and *Staphylococcus* groups. While the perinatal determinants surveyed in our multivariate approach explained only little about the staphylococcal load variability, we propose the maternal pre- and probiotic components in colostrum and breast milk to have been involved in the discerned differences. Moreover, the variability in starting proportions of fecal enterococci was associated with maternal urban residence, while the first month proportions, only, were related to the regional maternity hospitals and partial breastfeeding. Mode of delivery, however, reflected only in the first month *Enterococcus faecalis* proportions.

Infant early phenotypes - higher weight and lower ponderal index at first month, were associated with decreased proportions of group *Bacteroides-Prevotella*, which could suggest its protective role in infant development.

Chiefly, we would like to expose here the early mixed feeding practices and antibiotic treated health issues, for the manifestation of the most diverse and even extended shifts in microbial proportions related to the ‘evolutionary’ important taxa in infant gut. Later, during the first year, these two factors were further associated with higher weight gain deviation scores.

## Materials and Methods

### Ethics Statement

The study was conducted in accordance with the Helsinki declaration and was registered at https://clinicaltrials.gov/ct2/show/record/NCT01548313?term=MY+MILK&rank=1. The protocol was approved by the National Medical Ethics Committee of the Republic of Slovenia (32/07/10; 38/02/12). Written informed consent was obtained from all participating mothers. The recruitment proceeded in the years 2010–2011.

### Study Design and Participants

In this observational clinical study, healthy women in the first two trimesters of pregnancy (maximum 24 weeks gestation) were recruited during medical checks at their gynaecologists. We included expecting mothers from three Slovenian regions (Central, Pannonial and Mediterranean), who planned to breast-feed their babies for at least 6 weeks PP. Women suffering from acute or chronic infections or with an increased risk of a premature delivery were excluded from the study enrollment. Anthropometric data, demographic, lifestyle and clinical characteristics of mother-infant pairs were collected. Microbial analyses were performed on the DNA extracted from fecal samples from singleton infants.

### Clinical Data Collection and Anthropometric Measurements

The information on mother-infant demographic, clinical and environmental background was gathered from medical records and through interviews with research personnel at University Medical Centre Ljubljana (UMCL) and the attending physicians. Data on consumption of probiotics were obtained via self-administered questionnaire. Anthropometric data included maternal prepregnancy BMI (from medical records) and measurements of infant weight, length, and subscapular and triceps skinfolds. Data were acquired 0 to 3 days PP (3 days) at maternity hospitals and during UMCL visits at 4–5 weeks PP (30 days), 12–14 weeks PP (90 days), and at 12 months PP (1 year). BMI, PI, and BF% were calculated according to Slaughter *et al*.^49^. Infant’s expected growth was defined using the World Health Organisation’s (WHO) Child Growth Standards (2006)^50^. Sex standardized BMI-for-age Z-scores (not adjusted for a gestational age) were calculated by lmsGrowth Excel add-in^51^ based on the captured dataset (gender, age specified as days PP and BMI). The difference between BMI Z-score at the specific time point and at birth (BMI Z-score change) was estimated for each individual and served as an indicator of infant’s growth in the investigated time frame. Namely, according to WHO growth curves, theoretical, that is, anticipated child’s growth is submitted to its birthweight percentile (Z-score). Similarly, sex standardized WG Z-scores for an infant were calculated between the measured weights at the two selected time points. The percentage of newborns, following the expected growth, based on the Z-score change within the proposed cut-off values of −0.67 and + 0.67^52^, was also calculated.

### Fecal Sample Collection For Microbial Analysis

Infant fecal samples (approximately 2 g) were obtained at the maternity hospitals 2 to 4 days PP (3 days) and were immediately frozen at −20 °C. In the periods 4–5 weeks (30 days) and 12–14 weeks (90 days) PP mothers were instructed to collect fresh infant’s feces from a nappy in a provided sterile sampling container with a spoon attached to the lid. Until the ambulatory visit, fresh samples were kept refrigerated (4 °C) for not more than 24 hours and were immediately frozen (−20 °C) upon the arrival to UMCL. Within a month after the collection, fecal samples were transferred to the laboratory and were stored at −80 °C for up to a few months, until the DNA extraction and qPCR analyses.

### Dna Extraction And Qpcr Analyses

The protocol for fecal sample preparation and microbial DNA extraction has been in detail described elsewhere^53^. Briefly, after homogenization, lysis and sonication, samples were transferred to Maxwell 16 Tissue DNA Purification Kit (Promega) and the obtained 167 community DNA extracts served as a template for qPCR amplification of the following microbial groups: all bacteria, *Bacteroides-Prevotella* group, *Bifidobacterium* group, *Clostridium leptum* group, *Clostridium coccoides* group, *Enterobacteriaceae* group, *Enterococcus* group, *Enterococcus faecalis*, *Lactobacillus* group, *Lactobacillus gasseri*, *Staphylococcus* group, *Staphylococcus epidermidis* and *Streptococcus* group.

Maxwell DNA extracts from the respective microscopically enumerated pure-culture bacteria, as specified by Obermajer *et al*.^17^, but with the admixed treated fecal matrix, served as a basis for standard curve construction. QPCR analyses of fecal microbiota were performed by Mx3000 P instrument (Stratagene, La Jolla, CA, USA). For a single target gene, 6 assays were performed in a 96-well thermal block. Each assay included reactions with standard DNA dilutions (1:2) as well as reactions with 10-fold diluted sample DNA and non-template controls (NTCs), run in duplicates. Samples were distributed on plates randomly. The reactions were performed in a total volume of 20 µl, and the PCR mix consisted of KAPA SYBR Fast Master Mix (2x) Universal (KapaBiosystems, Boston, United States), 0.2 μM of each of the two oligonucleotide primers and 1 μL of DNA extract. Primers used were as listed by Obermajer *et al*.^17^ and Supplementary Table S2 ^54, 55^. The standard correlation chart between the observed cycles at the threshold fluorescence (Cq) and the estimated numbers of bacterial copies in the standard samples was processed by Mx3000 P software algorithms. Software settings based on the adaptive baseline correction and the amplification based threshold, as recommended by the manufacturer. The correlation coefficients (R^2^) and the amplification efficiencies (E) were calculated for different target reactions as presented in Table 3. Within a single target analysis the differences in the reaction E did not exceed 5.0% among the 6 runs compared, except for the *Enterobacteriaceae* group and *Staphylococcus* group, where the difference in the reaction E was 5.7% and 5.1%, respectively. Sample concentrations (copies per gram of feces) were calculated from the sample Cq interpolated into the standard plot. Limit of detection (LOD) for a single assay run was calculated from the equation of the standard curve while the last cycle number served as the cutoff point. Bacterial quantities extending below LOD for the specific assay were set to half of LOD value. The mean LODs for 6 assay runs, expressed as copies per reaction mix, are indicated in Table 3.

For the majority of samples, the difference between Cq values of technical replicates in different target reactions was ≤0.5. In the instances, where only one of the two replicates succeeded, a single value was used. For ‘all bacteria’ primer pair only, the NTCs crossed the threshold before the last cycle (30); except for four samples at day 3 and one sample at day 90, which were detected in the 10-fold NTC concentration area, quantification was conducted reliably. Melting curve analysis was performed at the end of each run to examine the curve profiles of sample replicates, standards and NTCs. At least five standard dilutions were included in each run, in different target reactions; the standards were covering a linear range from at least 2 to 5 logs.

### Statistical Analysis

Statistical analysis was carried out using SPSS version 23 (IBM SPSS, Chicago, IL, USA). For descriptive purposes, continuous variables were presented as medians (ranges), and frequencies (percentages) were used to describe categorical variables. Significance level was set at p < 0.05. The relative abundance of each bacterial group was determined as ratio between each group-specific qPCR assay (copy number estimate per gram) and all bacteria assay in the respective sample. Nonparametric Friedman test was used to assess longitudinal differences in absolute and relative abundances of microbial groups. Wilcoxon’s signed rank test was used in post hoc pair-wise comparisons and to assess the influence of feeding type (exclusive vs. partial breastfeeding). Holm’s method was used for control of type I error in post hoc tests. Associations of relative microbial abundances with clinical, environmental, anthropometric variables, and growth deviation scores were initially screened by bivariate analyses applying non-parametric Spearman’s rank correlation (continuous data) or Mann-Whitney U test (dichotomous data) to select predictor variables. The same approach was used to test for associations between various growth parameters and growth confounders. Growth deviation scores were calculated as BMI Z-score changes in individuals (i) between 1 month and birth (Z_1i_-Z_0i_), 3 months and birth (Z_3i_-Z_0i_), and between 12 months and birth (Z_12i_-Z_0i_); and as WG Z-scores for time periods between birth (0), 1, 3, and 12 months after birth as WG Z_(1–0)_, WG Z_(3–0)_, WG Z_(12–0)_, WG Z_(3–1)_, WG Z_(12–1)_, and WG Z_(12–3)_. Associations of the selected variables (p < 0.1) with relative microbial abundances (logit transformed) at specific time-point were then further evaluated by a multiple linear regression. Standardized effect size (differences in logit relative abundances) and significance (p < 0.05) for individual predictors were reported for the prevalent bacterial groups (detected in at least 30% of the samples). For the low prevalent bacterial groups (detected in less than 40% of the samples) multivariate logistic regression analysis was used on the dichotomized (detected/undetected) microbial abundance at specified time point. Associations between microbial groups in the forming gut communities were further investigated through the principal component analysis (PCA) of the relative abundance of 12 targeted microbial groups at 3 time points. Dimensionality of the problem was assessed by a scree plot. The results were presented as microbial group loadings on principal components (loading plot) and individual sample scores according to sampling time (scores plot).

### Data availability

All relevant data are within the paper and its Supplementary information file.

## Electronic supplementary material

Supplementary information file
