# Supplementary material for: Microbes in Infant Gut Development: Placing Abundance Within Environmental, Clinical and Growth Parameters
Source: Sci Rep. 2017 Sep 11;7:11230. doi: 10.1038/s41598-017-10244-x (PMC5593852; doi:10.1038/s41598-017-10244-x)
Supplement: Supplementary file 1 — Supplementary information file [file 41598_2017_10244_MOESM1_ESM.pdf]

# Microbes in Infant Gut Development: Placing Abundance Within Environmental, Clinical and Growth Parameters

**Tanja Obermajer[1*], Iztok Grabnar[2], Evgen Benedik[3] , Tina Tušar[1], Tatjana Robič Pikel[4], Nataša Fidler Mis[3], Bojana Bogovič Matijašić[1], Irena Rogelj[1]**

[1]Institute of Dairy Science and Probiotics, Biotechnical Faculty, University of Ljubljana, Slovenia,
[2]Faculty of Pharmacy, University of Ljubljana, Slovenia,
[3]Department of Gastroenterology, Hepatology and Nutrition, University Children's Hospital, University Medical Centre Ljubljana, Slovenia,
[4]Group of Anthropology, Department of Biology, Biotechnical Faculty, University of Ljubljana, 1000 Ljubljana, Slovenia

[*]For correspondence: E-mail tanja.obermajer@bf.uni-lj.si; Tel. (+386) 1 320 38 46; Fax (+386) 1 7214 074.

**Supplementary Table S1(a, b).** Multiple logistic regression analysis of the probability of detection for the low prevalent bacterial groups (detected in less than 40 % of samples). Predictor variables included in the regression model (yellow fill) were selected by *non-parametric* bivariate analyses of the associations with demographic, environmental, clinical, and anthropometric characteristics of the mother-infant pairs. Criterion for the inclusion in the model was ($p<0.1$). Only the data for bacterial groups with significant bivariate associations are presented. The color intensity indicates the strength and direction of the association, while numbers in the boxes show odds ratios. Irrelevant associations are indicated with black fill. Positive associations presented in light green fill ($p<0.05$) and negative associations in orange fill ($p<0.05$).

**a**

| TARGET GROUP | AGE AT SAMPLING - DAYS | GENDER- BOYS VS. GIRLS | GESTATIONAL AGE- FULL WEEKS | MODE OF DELIVERY- CESAREAN VS.VAGINAL | MATERNITY HOSPITAL- REGIONAL VS. MAIN | FEEDING TYPE 30- PARTIALLY VS. FULLY BREASTFED | FEEDING TYPE 90- PARTIALLY VS. FULLY BREASTFED | PROBIOTIC SUPPLEMENT- YES VS. NO | HEALTH BIRTH- UNHEALTHY VS. HEALTHY | HEALTH 30- UNHEALTHY VS. HEALTHY | HEALTH 90- UNHEALTHY VS. HEALTHY | ALLERGIES 0-6 MONTHS- YES VS. NO | RESIDENTIAL ENVIRONMENT- RURAL VS. URBAN | MATERNAL AGE- YEARS | MATERNAL EDUCATION- 5 STAGES FROM BASIC TO TERTIARY EDUCATION | MATERNAL SMOKING- YES VS. NO | PREGNANT WOMEN HEALTH- UNHEALTHY VS. HEALTHY | MATERNAL HEALTH AFTER BIRTH- UNHEALTHY VS. HEALTHY | MATERNAL BMI [kg/m$^2$] | WEIGHT BIRTH [g] | WEIGHT 30 [g] | WEIGHT 90 [g] | WEIGHT 1 YEAR [g] | BMI BIRTH [kg/m$^2$] | BMI 30 [kg/m$^2$] | BMI 90 [kg/m$^2$] | BMI 1 YEAR [kg/m$^2$] | PI BIRTH [kg/m$^3$] | PI 30 [kg/m$^3$] | PI 90 [kg/m$^3$] | PI 1 YEAR [kg/m$^3$] | BF% 30 | BF% 1 YEAR |
|---|---|---|---|---|---|---|---|---|---|---|---|---|---|---|---|---|---|---|---|---|---|---|---|---|---|---|---|---|---|---|---|---|---|
| *Lactobacillus gasseri* | 90 | | | | | | | | | | | | | | | | | | 1.30 | | | | | | | | | | | | | | |
| *Clostridium* cluster XIV | 30 | | | | | | | | | | | | | | | | | | 1.25 | | | | | | | | | | | | | | 1.57 |

| | | | | | | | | | | | | | | | | | | | | | | | | | | | | | | | | | |
|---|---|---|---|---|---|---|---|---|---|---|---|---|---|---|---|---|---|---|---|---|---|---|---|---|---|---|---|---|---|---|---|---|---|
| *Clostridium* cluster IV | 90 | | | | | 5.53 | | | | | | | | | | | | | | | | | | | | | | | | | | | |

**b**

| ⇐ TARGET GROUP | AGE AT SAMPLING - DAYS | BMI $Z_{1i}$ -$Z_{0i}$ | BMI $Z_{3i}$ -$Z_{0i}$ | BMI $Z_{12i}$ -$Z_{0i}$ | WG Z $_{(1-0)}$ | WG Z $_{(3-0)}$ | WG Z $_{(12-0)}$ | WG Z $_{(3-1)}$ | WG Z $_{(12-1)}$ | WG Z $_{(12-3)}$ |
|---|---|---|---|---|---|---|---|---|---|---|
| *Clostridium* cluster XIV | 30 | | | | | | | | | -0.30 |

**Supplementary Table S2.** Bacteria investigated by quantitative PCR analysis with their respective oligonucleotide primer pairs and the applied reaction conditions.

| Target organism | Target region | Product size (bp) | Annealing temp. (°C)/ time (sec) | Reference |
|---|---|---|---|---|
| *Enterococcus faecalis* | 16S rDNA | 360 | 57/30 | [54] |
| *Lactobacillus gasseri* | 16S-23S rDNA | 360 | 65/120 | [55] |

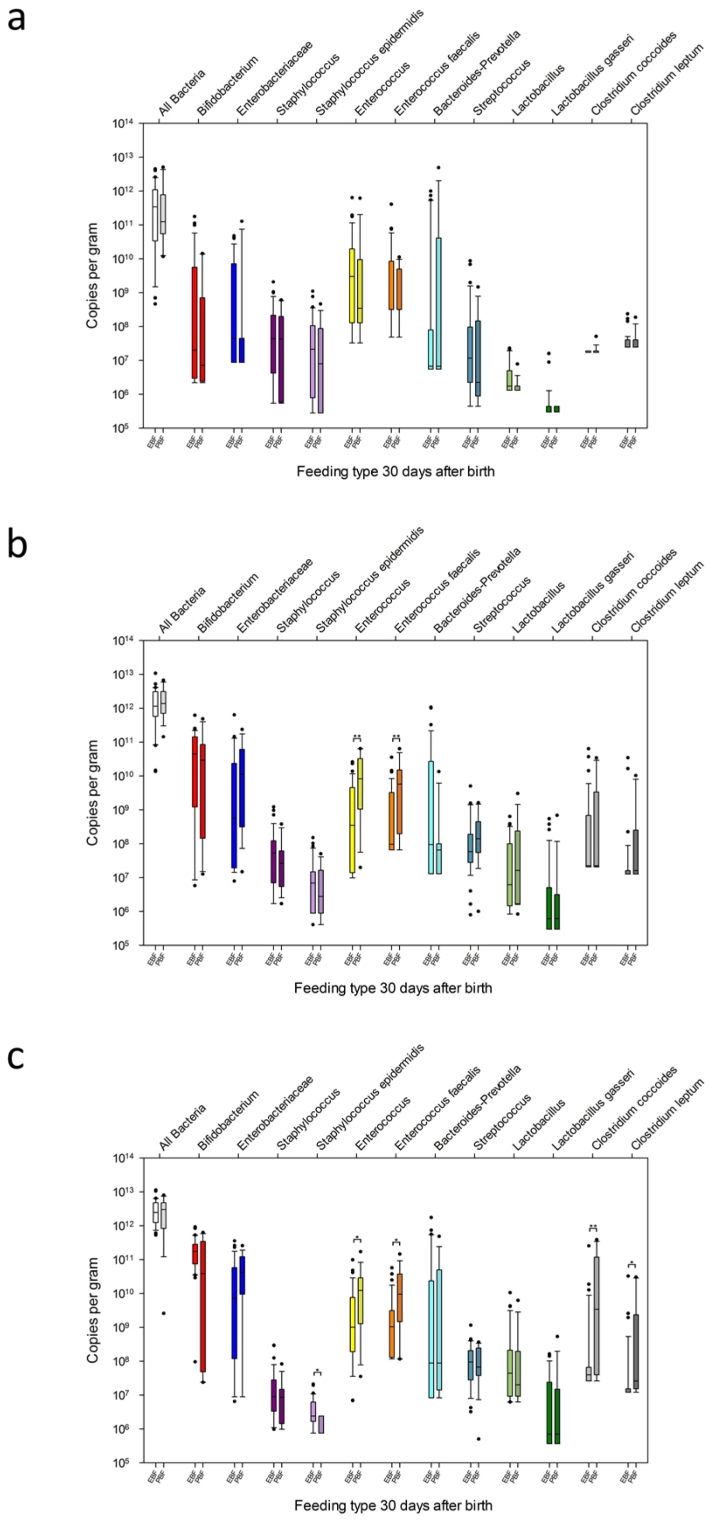


**Supplementary Figure S1.** Absolute abundance of fecal bacteria (copies per gram) in infants at 3 (a), 30 (b), and 90 (c) days after birth according to feeding type at 30 days (EBF – exclusively breastfed, PBF – partially breastfed). The box and whiskers plots represent the medians and interquartile ranges; error bars 10th and 90th percentiles, filled circles outliers. Asterisks denote significant differences (*p<0.05; **p<0.01).

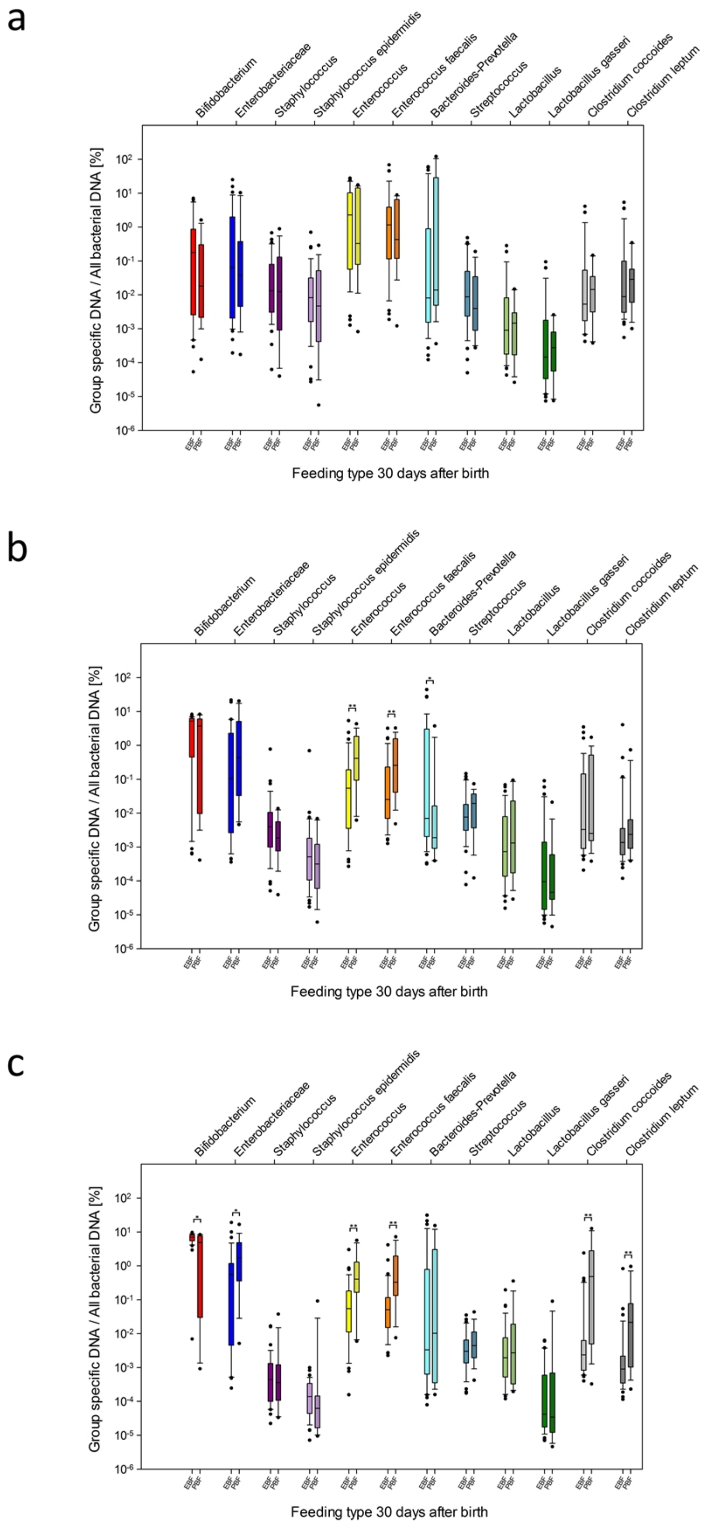


**Supplementary Figure S2.** Relative abundance of fecal bacteria (group specific DNA/all bacterial DNA) in infants at 3 (a), 30 (b), and 90 (c) days after birth according to feeding type at 30 days (EBF – exclusively breastfed, PBF – partially breastfed). The box and whiskers plots represent the medians and interquartile ranges; error bars 10th and 90th percentiles, filled circles outliers. Asterisks denote significant differences (*p<0.05; **p<0.01).
